# Immersive Technology for Cognitive-Motor Training in Parkinson’s Disease

**DOI:** 10.3389/fnhum.2022.863930

**Published:** 2022-05-09

**Authors:** Justin Lau, Claude Regis, Christina Burke, MaryJo Kaleda, Raymond McKenna, Lisa M. Muratori

**Affiliations:** ^1^College of Arts and Sciences, Stony Brook University, Stony Brook, NY, United States; ^2^Department of Physical Therapy, School of Health Professions, Stony Brook University, Stony Brook, NY, United States

**Keywords:** dual task, gaming, rehabilitation, gait, cognitive-motor interference, neurodegenerative disease, physical therapy

## Abstract

*Background:* Parkinson’s disease (PD) is a neurodegenerative disease in which the progressive loss of dopaminergic neurons (DA) leads to initially sporadic and eventually widespread damage of the nervous system resulting in significant musculoskeletal and cognitive deterioration. Loss of motor function alongside increasing cognitive impairment is part of the natural disease progression. Gait is often considered an automatic activity; however, walking is the result of a delicate balance of multiple systems which maintain the body’s center of mass over an ever-changing base of support. It is a complex motor behavior that requires components of attention and memory to prevent falls and injury. In addition, evidence points to the critical role of salient visual information to gait adaptability. There is a growing understanding that treatment for PD needs to address movement as it occurs naturally and walking needs to be practiced in more complex environments than traditional therapy has provided.

**Methods:**

In this single-blinded randomized-controlled pilot study, an immersive treadmill training was piloted to determine feasibility and preliminary efficacy on gait and cognition in people with PD. Eighteen participants with Hoehn and Yahr stages I-III PD were randomized to either an intervention or a waitlist control group. Following baseline data collection, the intervention group trained for 30 min, three times/week for 4 weeks on a split belt treadmill combined with a first-person immersive video game targeting visuospatial skills and working memory. Assessment was repeated after 4 weeks of training for the experimental group and 1-month after baseline for the control group. Primary motor outcomes were captured with the APDM Opal sensors during 6 MWT, TUG, and TUG Cognitive. Secondary outcomes of cognition were measured with the Montreal Cognitive Assessment (MoCA), Verbal Fluency (Fruit, Vegetable, and Animal) and the Symbol Digit Modality Test (SDMT). Within subject differences were calculated using the Wilcoxon Signed Ranked Test and between subject comparisons were analyzed using the Mann Whitney *U*-test.

**Results:**

This novel treadmill training program was well-tolerated with all participants in the intervention group completing 4 weeks of training three times a week without any adverse effects. After immersive cognitive motor training, the experimental group made clinically relevant improvements in gait speed and walking distance during the 6 MWT while members of the control group showed no change or decreased gait speed and walking distance over the 1-month trial. In addition, the experimental group demonstrated significant improvement for the TUG Cognitive (*p* = 0.05) and those changes were greater than the control group (between group *p* = 0.040). The experimental group also improved scores on MoCA (*p* = 0.007) and SDMT (*p* = 0.01) cognitive outcome measures while the control group did not.

**Conclusion:**

The use of immersive gaming technology to engage specific areas of cognition related to gait is feasible in PD. The treadmill training program paired with a customized interactive video game improved walking velocity in addition to non-significant but consistent improvements in other gait measures and cognitive performance in participants with early to mid-stage PD.

## Introduction

Parkinson’s disease (PD) leads to initially sporadic and eventually widespread damage of the nervous system resulting in significant musculoskeletal and cognitive deterioration (Del Tredici and Braak, 2012). Although symptoms vary, difficulty walking is a cardinal motor symptom of the disease. Clinically significant gait dysfunction occurs at some point in all persons with PD and 85% of individuals develop impairments within 3 years of diagnosis (Kang et al., 2005). The gait of patients with PD is typically marked by reduced speed, shortened stride length, and longer double support phase (Morris et al., 1994; Ebersbach et al., 1999; Sofuwa et al., 2005). In addition, gait dynamics are characterized by exaggerated stride-to-stride variability (Blin et al., 1990; Hausdorff et al., 1998; Schaafsma et al., 2003; Nasreddine et al., 2005; Baltadjieva et al., 2006) reflecting a disturbance in gait rhythmicity and an inconsistency of the locomotor pattern, increasing fall risk (Nakamura et al., 1996; Hausdorff et al., 2001; Schaafsma et al., 2003; Hausdorff, 2005). Loss of mobility is associated with reductions in quality of life, activities of daily living, and productivity in PD (Pickering et al., 2007; Forsaa et al., 2008; Muslimovic et al., 2008; Rahman et al., 2008).

Walking is not automatic; it involves cognitive areas of attention and memory (Holtzer et al., 2012), which are modulated by the dopaminergic system and known to be compromised in PD (Pagonabarraga and Kulisevsky, 2012). In PD, attentional demands may exceed available resources in tasks that depend on internal cues (Brown and Marsden, 1988). Several of the hallmark deficits in PD are due to changes in the frontal-striatal circuits and involve executive defects in planning, initiation, monitoring of goal-directed behaviors and working-memory. Visuospatial and memory deficits more representative of posterior cortical functioning are also present in persons with PD even without corresponding dementia (Pagonabarraga and Kulisevsky, 2012). In an experiment where motor and cognitive tasks were performed independently and combined in a dual task paradigm, individuals with PD showed distinct striatal recruitment that was not seen in single task performance or in the control participants. Results suggest that individuals with PD may have specific impairments of the cortical-striatal circuitry related to segregation needed to allow independence of motor and cognitive functions during dual tasking (Nieuwhof et al., 2017). There is a growing understanding that treatment needs to address movement as it occurs naturally and walking needs to be practiced in more complex environments than traditional therapy has provided (Moseley et al., 2005). Previous research has shown individuals with PD have greater deficits in gait under dual-task conditions than their healthy peers. Gait velocity and variability increases while cognitive performance on standardized tests decreases under dual-task conditions (O’Shea et al., 2002; Lord et al., 2010; Plotnick et al., 2011). In their review of this literature, Kelly et al. (2012) point out that the frequency of multitasking and the resulting impairments creates a pressing need for therapeutic interventions that address dual-task gait deficits in PD.

The use of treadmills in gait rehabilitation in PD patients improves gait performance (Miyai et al., 2000; Pohl et al., 2003; Herman et al., 2007; Bello et al., 2008, 2010). The repetitive, regulated walking on a treadmill improves spatiotemporal gait parameters and those improvements are maintained in PD (Mehrholz et al., 2016). Importantly, walking on a treadmill allows patients to increase walk intensity (increasing time on task) in a safe manner. However, traditional treadmill walking has a number of limitations. Standard treadmill training has minimal real-world sensory information necessary for motor learning. Previous work in PD (Rochester et al., 2010) and stroke (Peters et al., 2015) have shown the importance of providing salient sensory information, including combined proprioceptive and visual cues, to create long-term improvement in gait. Visuospatial information, particularly that created by optic flow during walking, is specifically important in healthy, adaptable gait control (Bruce et al., 1996; Patla et al., 2004; Mukherjee et al., 2011; Chien et al., 2014). Similarly, in PD, visual control appears to be critical during simple (Azulay et al., 1999) and complex (Vitório et al., 2013) walking.

Another restriction of treadmills typically used for rehabilitation are that they change speed and inclination only after user input explicitly cueing the performer of an upcoming adjustment. Step length variability (left-right step length variation) is likewise limited by a single belt moving under the walker. A split-belt treadmill, comprised of two belts as opposed to the conventional one belt, allows for users to drive each foot independently of the other and allows clinicians to target left-right asymmetries. A training regimen that utilizes both a split belt treadmill and an external game controller that links an immersive visual experience to movement of the treadmill, may provide another option for training motor adaption with varying amounts of complexity while also providing the benefits of ecologically valid visual information. This pilot study examines the feasibility and preliminary efficacy of such a novel immersive treadmill training program to improve automaticity of gait by addressing both the movement deficits and cognitive components critical to walking and walking adaptation by providing continuous, salient sensorimotor information.

## Materials and Methods

This study had approval from the Stony Brook University ethics committee (00646/https://clinicaltrials.gov/ct2/show/NCT01917903) and all participants gave written informed consent in accordance with the Declaration of Helsinki prior to data collection. Testing took place in the Rehabilitation Research and Movement Performance Laboratory, School of Health Professions, Stony Brook University.

### Participants

Twenty-two participants with PD were recruited for inclusion in this study. Inclusion criteria included: confirmed diagnosis by a movement disorder neurologist; 21–79 years of age, ability to walk 15 min with or without an assistive device, and ability to understand all study procedures and sign informed consent documents. Exclusion from the study was based on not meeting the inclusion criteria, secondary orthopedic or neurological injury or disease that affected gait or balance, visual or vestibular deficits that impair walking, and diagnosis of a cognitive impairment unrelated to PD. Randomization was performed using a random number generator for five bins of 6, 4, 4, 4, and 4 participants recruited (i.e., randomization occurred after recruitment of the initial 6, and each set of 4 participants afterward). One participant was over 80 years of age after initial consent and randomization to the experimental group but prior to completing data collection and was removed from further inclusion. Two participants withdrew following randomization to the waitlist group but prior to any data collection. One participant allocated to the experimental group demonstrated significant cognitive difficulties. After follow-up with neurology a secondary cognitive impairment was diagnosed. Training was completed but data was not included for this participant in analysis. Eighteen participants with PD, modified Hoehn and Yahr stages 1–3 (Goetz et al., 2004) randomly allocated to the experimental (*N* = 9) or waitlist control group (*N* = 9) completed all testing and are included in all data analyses. Demographic data for individual participants are provided in Table 1.

**TABLE 1:** Participant demographics.

Participant ID	Sex	Age	H&Y stage	PD medications	Baseline gait velocity	Baseline MoCA
E1	F	68	1	Y	0.93	27
E2	M	75	2	Y	0.96	26
E3	M	57	2	Y	1.02	27
E4	F	57	2	Y	0.99	29
E5	M	71	3	Y	0.95	23
E6	M	59	2	N	0.94	28
E7	M	64	1.5	Y	1.09	23
E8	M	48	2	Y	1.04	25
E9	F	70	2.5	Y	0.69	26
Mean (SD)	**6M/3F**	**64 (9)**	**2 (0.56)**	**8/9**	**0.96 (0.11)**	**26 (2)**
C1	M	78	2	Y	0.72	23
C2	F	69	2	Y	1.05	25
C3	M	73	3	Y	0.92	26
C4	M	79	2.5	Y	0.83	18
C5	M	65	1	Y	1.17	29
C6	F	72	2	Y	0.87	24
C7	F	72	1.5	Y	1.17	28
C8	M	66	2	Y	1.01	30
C9	M	67	1	Y	1.08	30
Mean (SD)	**6M/3F**	**71 (5)**	**1.9 (0.65)**	**9/9**	**0.98 (0.16)**	**26 (4)**

*Sex, Age, Hoehn and Yahr (H&Y) stage, use of PD medication, baseline velocity, and baseline MoCA score for individual participants in each group. Participants in the Experimental group (E1–E9) and Control group (C1–C9) were equivalent at baseline (p > 0.05 for all comparisons). There was only one participant (E6) that was not taking medication (dopamine, dopamine agonists, anticholinergics, COMT or MAO inhibitors) for PD. Means and SD are in bold.*

### Procedures

All assessments and training were performed with participants ON medication. Following randomization, baseline data was collected by two assessors (CB and MK) blinded to group allocation. Participants completed standing and walking assessments while wearing APDM Opal (Portland, OR) body worn inertial sensors on both wrists, mid-chest, lumbar spine, and both feet. Motor tasks were completed in the same order for each session and included the 6-min walk test (6 MWT), Timed Up and Go (TUG) (Podsiadlo and Richardson, 1991) and TUG Cognitive (performing the TUG while subtracting 3 from a randomly selected number in the 90s) (TUG Cognitive) (Schumway-Cook et al., 2000). In addition, participants were administered the Symbol Digit Modalities Test (SDMT) (Smith, 1982), Montreal Cognitive Assessment (MoCA), and Tests of Verbal Fluency Fruits, Vegetables, and Animals (TVF_F, TVF_V, and TVF_A, respectively) (Battig and Montague, 1969). All measures were repeated at the end of 4 weeks of training for the experimental group and after 1-month of usual activity for the control group.

**FIGURE 1:**
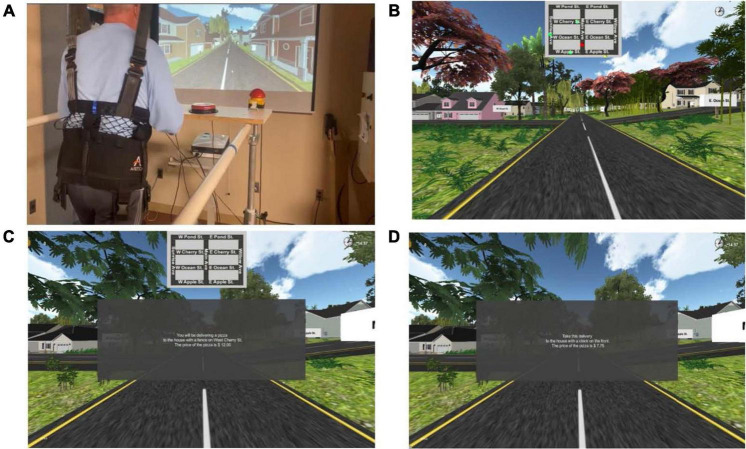
Experimental set-up. **(A)** Experimental set-up including a split-belt treadmill, overhead safety harness (no bodyweight support), large button controllers (to turn in the game left or right and reverse directions and to select the correct change following a delivery), projector and screen. A separate computer work station to the right of the participant allowed the research clinician to control treadmill speed and inclination. Both the treadmill and the computer workstation had emergency stop capability. **(B)** Level one of the immersive game: Participants are shown a map with the location of the participant in the game (red dot), target location for delivery (yellow dot), and location of three thieves moving around the game (green dots). **(C)** Level two of the immersive game: Participants are shown the map of the town but are no longer provided with dots to show where they are, where they need to go, or where the thieves are located. The dialogue in the center of the screen is a typical starting instructional prompt. Level three has the same type of prompt but the map is no longer visible. **(D)** Level four of the immersive game: similar to level three, the map is not visible in level four. The dialogue in the center shows that the instructional prompt now has a more complex price and the calculation required at delivery is similarly more difficult.

Participants in the experimental group completed 360 min of training (30 min per session, 3 times per week × 4 weeks) using a novel, immersive rehabilitation program developed to target gait and cognition. Executive function and visuospatial attention tasks were specifically included as they have been previously described as critical components of walking (Amboni et al., 2013). Training consisted of walking on a split-belt treadmill (Woodway, Inc., Waukesha, WI, United States) connected to a computer running a proprietary first-person gaming system written using C + coding^1^ and projected on a large screen to include the entire visual field of the participant while they walked on the treadmill. Game controls were positioned on a board positioned in front of the participant and affixed to the treadmill and included three large push buttons (Ablenet, Inc., Roseville, MN, United States) to control forward/reverse, right, and left turns in the game (see Figure 1). Maximum and minimum inclination of the treadmill and speed of each leg—adjusted independently to allow for individualized attention to gait symmetry—was set by the training clinicians (RM and LM). Treadmill speed was set initially using the participant’s baseline gait speed and then adjusted during subsequent sessions to maintain an optimal challenge (Guadagnoli and Lee, 2004) for each subject. Each session consisted of two 15-min games, with a rest as needed in-between, so that participants completed 30 min of training each session.

The experimental group trained three times a week for 4 weeks. An overhead harness was used for safety but did not provide any bodyweight support. The game involved walking through a virtual town to make as many deliveries as possible in 15 min. Participants self-initiated the game by pushing any of the control buttons. Throughout the sessions, participants were able to view their score as well as a clock counting down from 15 min. The level of game play became progressively more difficult each week as follows:

• **Week 1**—Game interface included a map that showed the location of the participant (red dot), the target location (yellow dot), and the location of the thieves moving around the town (see Figure 1B).
• **Week 2**—Game interface showed a map of the town but the location of the participant, target location, and location of the thieves was no longer included (see Figure 1C).
• **Week 3**—The map was no longer shown, requiring the participant to remember the location of streets and houses previously visited as well as visual scan for thieves.
• **Week 4**—The map is still not present, an additional thief is added, and the prices of the delivered item and the amount paid by the customer is more complex, requiring greater calculations (see Figure 1D).

The system recorded treadmill speed of each leg, minimum and maximum inclines, game scores, total deliveries made, and calculation errors for each session. These measures were not part of the efficacy analyses but will be investigated for clinical utility in the future.

### Data Analyses

All APDM sensor data were processed using Mobility Lab software (Version 2). Statistical analyses were completed with SPSS Version 26 (IBM, Armonk NY). Demographic data was compared using independent *t*-tests assuming equal variance. We used a Wilcoxon Signed Rank test to examine differences in motor (gait speed, 6 MWT distance, TUG, TUG Cognitive) and cognitive (MoCA, SDMT, TVF) task performance from baseline to 1-month post-training (**E**xperimental) or 1-month of usual activity (**C**ontrol) within subjects. For between subject comparisons, the Mann Whitney *U*-tests was used. We present graphic representations of all individual data points and provide descriptions of differences in relation to clinically meaningful changes observed.

## Results

### Feasibility

All participants in the experimental group completed 4 weeks of training without any adverse events. There was 94.4% (34/36 trials) completion of the training trials with two sessions missed due to mechanical issues with the treadmill rather than any participant difficulties. Two of nine participants required seated rests of 5 min between the two 15-min games while the other seven chose short standing rest periods of less than 5 min. All 34 training sessions lasted less than 1 h. Demographic information is shown in Table 1. Participants in the experimental group were not significantly different than the control group for any of baseline measures.

### Motor Outcomes

**FIGURE 2:**
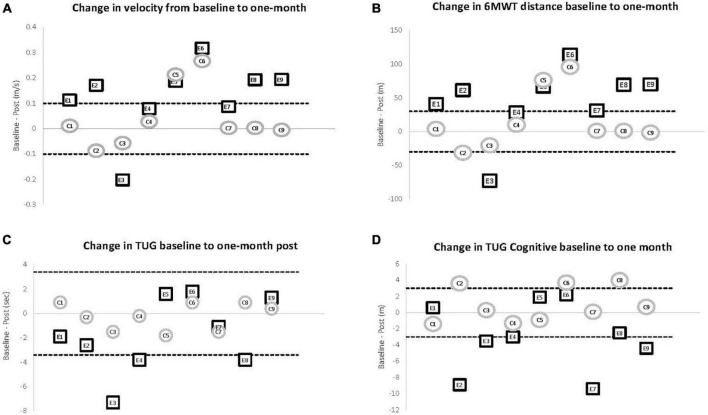
Motor outcomes comparing baseline to 1 month post. Individual change scores from baseline to 1-month for participants in the experimental (E1–E9) and control (C1–C9) groups. The solid line at 0 represents no change at the 1-month post assessment (zero change). The dashed lines show the level reported for clinically meaningful change in the respective variable. **(A)** Change in gait velocity. An increase of 0.14 m/s (top dashed line) or more represents a clinically meaningful change in velocity for individuals with PD. Six of the nine participants in the experimental group improved at or past the dashed line while only two of the nine control participants demonstrated that amount of improvement. **(B)** Change in distance for the 6 MWT. The dashed line represents a change of 30 m as an increase (top dashed line) of that amount has been shown to represent a clinically meaningful change in the 6 MWT for individuals with PD. Seven of the nine participants in the experimental group improved at or past the dashed line while only two of the nine control participants demonstrated that amount of improvement. **(C)** Change scores for TUG. A decrease of 0.3 m/s (bottom dashed line) has been shown to represent a clinically meaningful change on this outcome measure. Three of the nine participants in the experimental group improved at or past the dashed line while none of the control participants demonstrated meaningful improvement. **(D)** Change scores for TUG Cognitive The dashed line represents a change of 3 s in either direction and was chosen to match the criteria for the TUG. Three of the nine participants in the experimental group improved while none of the control participants demonstrated that amount of improvement.

The within and between subject analysis did not show significant differences for any of the motor performance measures except the TUG Cognitive. However, distance and gait speed measured during the 6 MWT trended toward significance for the experimental group with eight of the nine participants increasing speed and walking further post-training (*p* = 0.086). Small and moderate clinically meaningful difference of 0.06 and 0.14 m/s, respectively (Hass et al., 2014), were used to compare the outcomes among the two groups. One month from baseline, six participants in the experimental group showed moderate gains and another two participants had small but meaningful improvement. In the control group, only two participants had clinically meaningful changes in velocity, both in the moderate range (see Figure 2A). Similarly, considering an increase of 30 m to represent a clinically important change in distance traveled during the 6 MWT (Bohannon and Crouch, 2016), seven of nine people in the experimental group improved and one other increased distance 28.5 m, approaching the minimally clinically important difference. Only two control participants exceeded a 30 m increase in walking distance (see Figure 2B). While the 6 MWT is explicitly linked to gait speed, it is important to demonstrate that the change in speed resulted in a meaningful change in distance walking over the duration of the test. Indeed, one person who did not quite meet the criteria for a moderately clinically meaningful change in velocity did increase walking distance by more than 30 m.

Comparison of the baseline to 1-month testing of the TUG and TUG Cognitive demonstrated that most participants in the experimental group could incorporate the increase in gait speed into functional tasks. Interestingly, it was the TUG Cognitive, an outcome measure that directly measures combined cognitive and motor performance, that showed statistically significant improvement within the experimental group (*p* = 0.05) but not the control group (*p* = 0.37) resulting in a between group difference on the Mann-Whitney *U*-test (*p* = 0.040). Indeed, six participants in the experimental group showed greater improvement than anyone in the control group (see Figures 2C,D).

### Cognitive Outcomes

**FIGURE 3:**
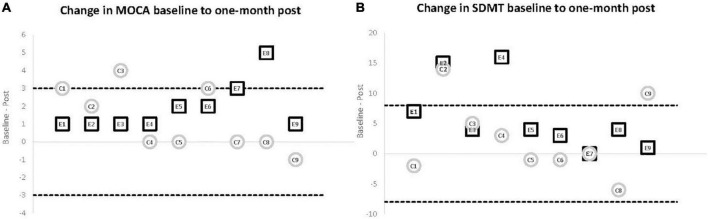
Cognitive outcomes comparing baseline to 1 month post. Individual change scores from baseline to 1-month for participants in the experimental (E1–E9) and control (C1–C9) groups. The solid line at 0 represents no change at the 1-month post assessment (zero change). The dashed lines show the level reported for clinically meaningful change in the respective variable. **(A)** Change scores for MoCA. The dashed line represents a change of 3-points in either direction. An increase (top dashed line) of that amount has been shown to represent a clinically meaningful change on this outcome measure. While the experimental group showed a consistent, significant improvement (*p* = 0.007) with all nine participants improving on the follow-up test, only two improved by 3 or more points. The control participants did not demonstrate a significant change and showed a more varied pattern, with four participants improving and the other five remaining the same or worse at 1-month testing. **(B)** Change scores for SDMT. The dashed line represents a change of 8-points in either direction. An increase (top dashed line) of that amount has been shown to represent a clinically meaningful change on the SDMT. While the experimental group showed a consistent, significant improvement (*p* = 0.01) with eight of nine participants improving on the follow-up test, both the experimental and control groups had only two participants improve 8 or more points. The control participants did not demonstrate a significant change and four of the control participants had a poorer performance at 1-month testing.

Although change in cognitive performance was not a main outcome in this pilot study, the comparison from baseline to post-training (experimental) and 1-month post usual activity (control) revealed significant differences in outcomes. While neither group showed significant improvements in any of the verbal fluency tests, the experimental group showed significant improvement in both the MoCA (*p* = 0.007) (Figure 3A) and SDMT (*p* = 0.01) (Figure 3B) with all 9 participants improving on the MoCA and 8 of 9 improving on the SDMT. In the control group, baseline to post change in the MoCA approached significance (*p* = 0.08) but with only 4 participants showing improved scores, 4 showing no change and the final participant showing decline. Similarly, control participants did not show overall improvement in the SDMT (*p* = 0.40) with 4 participants improving, 1 participant showing no change, and 4 participants with poorer performance after 1-month of usual activity (see Figure 3).

## Discussion

Leveraging technology to treat PD is not new. Deep brain stimulation is a well-established tool to improve motor performance even when medication is not effective (Groiss et al., 2009). In addition, there is increasing literature demonstrating the benefits of wearable sensors for the assessment and management of PD symptoms (Rovini et al., 2017). However, the use of technology for rehabilitation has not always considered real-life environments during functional mobility training. The principle of ecological validity for rehabilitation is that gait training should not take place only under optimal conditions in a therapeutic gym; rather, walking should be practiced in increasingly complex and variable environments with appropriate sensory information (Hallett and Poewe, 2008). Previous work has demonstrated the potential of dual task training to improve gait in PD (Yang et al., 2019) based on known cognitive motor interaction deficits (Yogev et al., 2005). This pilot study demonstrated the feasibility and preliminary efficacy of an immersive training with complex cognitive engagement and multisensory information processing. Importantly, changes in motor behaviors reached clinically meaningful levels after twelve 30-min sessions, well within the scope of traditional clinical practice.

The use of a treadmill with clinician interface to train under an optimal challenge point framework (Guadagnoli and Lee, 2004) was chosen as it provided a clinically feasible set-up for rehabilitation. Previous work in PD has shown that gait training with a treadmill can improve stride length and foot clearance (Bello et al., 2014) and lead to clinically meaningful improvements in gait velocity (Mehrholz et al., 2016). We found similar improvements in gait velocity and corresponding walking distance over 6 min. Herman et al. (2007) found that progressive, intensive treadmill training over a 6-week period improved both motor skills and quality of life and that these improvements were maintained several weeks after training had ceased. The mechanism underlying the improvement from use of the treadmill is not confirmed but likely includes neuroplastic changes from the intensity of training from the large volume of stepping that can be practiced (Hirsch et al., 2016) and increased sensory cues from the movement of the treadmill (Bello et al., 2010). Interestingly, there has been some suggestion that the individual with PD most likely to benefit from treadmill training is someone with intact cognition (Earhart and Williams, 2012). In this study, cognition was explicitly targeted and shown to improve with 4-weeks of individualized treadmill training.

The use of a split-belt treadmill has been recommended specifically for PD because of the laterality common in presentation (Seuthe et al., 2019). In this study, training clinicians were able to independently regulate the speed of the right and left treadmill belts to work on gait symmetry even though it was not an outcome in our preliminary work. While multiple studies have shown that individuals with PD can adapt to split-belt walking, some work suggests no improvement in coordination post-training (Nanhoe-Mahabier et al., 2013) while other studies indicate that carryover to overground walking may be specific to whether the more impaired or less impaired leg is manipulated on the treadmill (Fasano et al., 2016). More work is needed in this area to determine the potential use of split-belt vs. traditional treadmill training in PD.

While motor symptoms are the hallmark of PD, the presence of cognitive deficits is a well-established non-motor impairment (Meireles and Massano, 2012) that worsens with disease duration (Dubois et al., 2007) and may appear prior to the onset of motor symptoms (Pont-Sunyer et al., 2015). Cognitive training in PD has also shown specific benefits for targeted areas of treatment, including global cognition, attention, and verbal memory (Orgeta et al., 2020). The vast majority of these studies isolated cognition from motor impairments but there is recent work addressing both movement and cognition through enriched environment (Wang et al., 2021) and dual task training (for review, see De Freitas TB MS et al., 2020). Similar to these studies, we found that individuals with PD were able to improve gait speed and functional gait (TUG and TUG Cognitive). However, we additionally showed that integrating targeted cognitive training, including executive control, visuospatial working memory, and attention, during treadmill training resulted in improved cognitive scores as well. Combining cognitive and motor training into a single intervention represents a new way of addressing the complex deficits in PD. First, the treadmill walking in and of itself may lead to exercise-induced neuroplastic changes in motor and cognitive processing (Petzinger et al., 2013). While aerobic capacity was not measured, training combined two bouts of 15-min walking at a challenging pace for a 30-min exercise session. Second, the use of an immersive, visually engaging treadmill allowed for repeated training of areas of cognition important to gait to occur simultaneously with walking. The different game levels each week was intentionally directed at maximizing the cognitive challenge. The effect was that this training addressed both the motor and non-motor symptoms of PD.

Interestingly, both the trainers and assessors noted clear phenotypic differences in participants as they performed the assessments and/or training. PD is a heterogenous disease often viewed as a movement disorder with three motor types: Tremor-dominant; akinetic-rigid or postural instability with gait difficulty; or indeterminate (Luo et al., 2019; Wojtala et al., 2019; Markello et al., 2021). The clinical spectrum of PD encompasses many non-motor domains like cognition and autonomic function. In addition to motor phenotypes, PD has been characterized by the severity of non-dopaminergic factors in combination with motor complications. Cluster analysis showed four distinct subtypes, each with multiple motor and non-motor characteristics with groups varying by symptom severity (van Rooden et al., 2011). Biomarker studies show different physiological changes between tremor- dominant PD and non-tremor-dominant PD (Marras and Chaudhuri, 2016), but clinically, individuals rarely remain in one subtype throughout their course of disease (Nutt, 2016). While we did not classify participants, it may have been that the consistent outlier in our findings (participant E3) represented a different subtype. The preliminary findings suggest that stratifying by subgroup may provide more precise treatment response information and be beneficial to rehabilitation treatment planning.

### Limitations

This study was limited by the small number of participants in each group. Of the 22 participants recruited, 18 were included and completed all the testing. The withdrawal of two participants after randomization to the waitlist control group suggests that there is a desire for individuals with PD to engage in regular training with new technology. This pilot was structured to provide proof of concept and feasibility of an immersive cognitive motor training program and, therefore, was not powered to make conclusive statements about long-term efficacy. In addition, although the training progression was clinician-driven based on perceived challenge points, the study would have benefited from collection of participant perceptions of exertion (e.g., monitoring heartrate or use of a Borg scale during targeted sessions).

Acceptance of new technology is a critical component of implementation science. It is important to gather more information about the perceptions of the participants and clinicians who might use this training program in clinics. Further data analysis will include quality of life measures and focus groups with qualitative assessment of the experience of the participants who completed the four weeks of training.

Finally, this study utilized a split-belt treadmill to access the ability of the participants to maintain continuous therapeutic walking speeds on separate belts and to provide clinicians a further avenue of individualizing treatment. However, it is unclear from the current data set whether a split-belt treadmill provides any benefits over a standard treadmill and, importantly, as a split-belt treadmill is less clinically available it may make such training less feasible for implementation.

## Data Availability Statement

The raw data supporting the conclusions of this article will be made available by the authors, without undue reservation.

## Ethics Statement

The studies involving human participants were reviewed and approved by the Stony Brook University Institutional Review Board. The patients/participants provided their written informed consent to participate in this study.

## Author Contributions

LM conceptualized the research project. LM, JL, CR, CB, MK, and RM executed. RM and LM designed and executed the statistical analysis. JL, CR, and LM organized and wrote all drafts of the manuscript. RM, CB, and MK did the review and edits. All authors contributed to the article and approved the submitted version.

## Conflict of Interest

The authors declare that the research was conducted in the absence of any commercial or financial relationships that could be construed as a potential conflict of interest.

## Publisher’s Note

All claims expressed in this article are solely those of the authors and do not necessarily represent those of their affiliated organizations, or those of the publisher, the editors and the reviewers. Any product that may be evaluated in this article, or claim that may be made by its manufacturer, is not guaranteed or endorsed by the publisher.

## Footnotes

1
www.unity.com

## References

[B1] Amboni M. Barone P. Hausdorff J. (2013). Cognitive contributions to gait and falls: evidence and implications. *Mov. Disord.* 28 1520–1533. 10.1002/mds.25674 24132840 PMC4119872

[B2] Azulay J. P. Mesure S. Amblard B. Blin O. Sangla I. Pouget J. (1999). Visual control of locomotion in Parkinson’s disease. *Brain* 122 111–120. 10.1093/brain/122.1.111 10050899

[B3] Baltadjieva R. Giladi N. Gruendlinger L. Peretz C. Hausdorff J. M. (2006). Marked alterations in the gait timing and rhythmicity of patients with de novo Parkinson’s disease. *Eur. J. Neurosci.* 24 1815–1820. 10.1111/j.1460-9568.2006.05033.x 17004944

[B4] Battig W. F. Montague W. E. (1969). Category norms of verbal items in 56 categories a replication and extension of the Connecticut category norms. *Journal of Experimental Psychology.* 80 1–46. 10.1037/h0027577

[B5] Bello O. Marquez G. Camblor M. Fernandez-Del-Olmo M. (2010). Mechanisms involved in treadmill walking improvements in Parkinson’s disease. *Gait Posture* 32 118–123. 10.1016/j.gaitpost.2010.04.015 20452773

[B6] Bello O. Sanchez J. A. Fernandez-del-Olmo M. (2008). Treadmill walking in Parkinson’s disease patients: adaptation and generalization effect. *Mov. Disord.* 23 1243–1249. 10.1002/mds.22069 18464281

[B7] Bello O. Sanchez J. A. Vazquez-Santos C. Fernandez-Del-Olmo M. (2014). Spatiotemporal parameters of gait during treadmill and overground walking in Parkinson’s disease. *J. Parkinsons Dis.* 4 33–36. 10.3233/JPD-130251 24496097

[B8] Blin O. Ferrandez A. M. Serratrice G. (1990). Quantitative analysis of gait in Parkinson patients: increased variability of stride length. *J. Neurol. Sci.* 98 91–97. 10.1016/0022-510x(90)90184-o 2230833

[B9] Bohannon R. W. Crouch R. (2016). Minimal clinically important difference for change in 6-minute walk test distance of adults with pathology: a systematic review. *J. Eval. Clin. Pract.* 23 377–381. 10.1111/jep.12629 27592691

[B10] Brown R. G. Marsden C. D. (1988). Internal versus external cues and the control of attention in Parkinson’s disease. *Brain* 111 323–345. 10.1093/brain/111.2.323 3378139

[B11] Bruce V. Georgeson M. A. Green P. R. (1996). *Visual Perception: Physiology, Psychology, and Ecology*, 3rd Edn. Hove: Psychology Press.

[B12] Chien J. H. Eikema D.-J. A. Mukherjee M. Stergiou N. (2014). Locomotor sensory organization test: a novel paradigm for the assessment of sensory contributions in gait. *Ann. Biomed. Eng.* 42 2512–2523. 10.1007/s10439-014-1112-7 25224076 PMC4241158

[B13] De Freitas TB MS P. T. Leite Phw B. S. Doná F PhD P. T. Pompeu Je PhD P. T. Swarowsky A PhD P. T. Torriani-Pasin C PhD P. T. (2020). The effects of dual task gait and balance training in Parkinson’s disease: a systematic review. *Physiother Theory Pract.* 36 1088–1096. 10.1080/09593985.2018.1551455 30501424

[B14] Tredici K. D. Braak H. (2012). Lewy pathology and neurodegeneration in premotor Parkinson’s disease. *Mov. Disord.* 27 597–607. 10.1002/mds.24921 22508278

[B15] Dubois B. Burn D. Goetz C. Aarsland D. Brown R. G. Broe G. A. (2007). Diagnostic procedures for Parkinson’s disease dementia: recommendations from the Movement Disorder Society task force. *Mov. Disord.* 22 2314–24. 10.1002/mds.21844 18098298

[B16] Earhart G. M. Williams A. J. (2012). Treadmill training for individuals with Parkinson disease. *Phys. Ther.* 92 893–897. 10.2522/ptj.20110471 22539229 PMC3386513

[B17] Ebersbach G. Sojer M. Valldeoriola F. Wissel J. Müller J. Tolosa E. (1999). Comparative analysis of gait in Parkinson’s disease, cerebellar ataxia and subcortical arteriosclerotic encephalopathy. *Brain* 122 1349–1355. 10.1093/brain/122.7.1349 10388800

[B18] Fasano A. Schlenstedt C. Herzog J. Plotnik M. Rose F. E. M. Volkmann J. (2016). Split-belt locomotion in Parkinson’s disease links asymmetry, dyscoordination and sequence effect. *Gait Posture* 48 6–12. 10.1016/j.gaitpost.2016.04.020 27477701

[B19] Forsaa E. B. Larsen J. P. Wentzel-Larsen T. Herlofson K. Alves G. (2008). Predictors and course of health-related quality of life in Parkinson’s disease. *Mov. Disord.* 23 1420–1427. 10.1002/mds.22121 18512757

[B20] Groiss S. J. Wojtecki L. Südmeyer M. Schnitzler A. (2009). Deep Brain Stimulation in Parkinson’s Disease. *Ther. Adv. Neurol. Disord.* 2 20–28. 10.1177/1756285609339382 21180627 PMC3002606

[B21] Guadagnoli M. A. Lee T. D. (2004). challenge point: a framework for conceptualizing the effects of various practice conditions in motor learning. *J. Motor Behav.* 36 212–224. 10.3200/jmbr.36.2.212-224 15130871

[B22] Hallett M. Poewe W. (2008). *Therapeutics of Parkinson’s Disease and Other Movement Disorders.* Hoboken, NJ: Wiley-Blackwell.

[B23] Hass C. J. Bishop M. Moscovich M. Stegemöller E. L. Skinner J. Malaty I. A. (2014). Defining the clinically meaningful difference in gait speed in persons with Parkinson disease. *J. Neurol. Phys. Ther.* 38 233–238. 10.1097/npt.0000000000000055 25198866

[B24] Hausdorff J. M. (2005). Gait variability: methods, modeling and meaning. *J. NeuroEng. Rehabil.* 2 19. 10.1186/1743-0003-2-19 16033650 PMC1185560

[B25] Hausdorff J. M. Cudkowicz M. E. Firtion R. Wei J. Y. Goldberger A. L. (1998). Gait variability and basal ganglia disorders: Stride-to-stride variations of gait cycle timing in Parkinson’s disease and Huntington’s disease. *Mov. Disord.* 13 428–437. 10.1002/mds.870130310 9613733

[B26] Hausdorff J. M. Rios D. A. Edelberg H. K. (2001). Gait variability and fall risk in community-living older adults: a 1-year prospective study. *Arch. Phy. Med. Rehabil.* 82 1050–1056. 10.1053/apmr.2001.24893 11494184

[B27] Herman T. Giladi N. Gruendlinger L. Hausdorff J. M. (2007). Six weeks of intensive treadmill training improves gait and quality of life in patients with Parkinson’s disease: a pilot study. *Arch. Phys. Med. Rehabil.* 88 1154–1158. 10.1016/j.apmr.2007.05.015 17826461

[B28] Hirsch M. A. Iyer S. S. Sanjak M. (2016). Exercise-induced neuroplasticity in human Parkinson’s disease: WHAT is the evidence telling us? *Parkinson. Relat. Disord.* 22 S78–S81. 10.1016/j.parkreldis.2015.09.030 26439945

[B29] Holtzer R. Wang C. Verghese J. (2012). The relationship between attention and gait in aging: facts and fallacies. *Motor Control.* 16 64–80. 10.1123/mcj.16.1.64 22402221 PMC3471155

[B30] Kang G. A. Bronstein J. M. Masterman D. L. Redelings M. Crum J. A. Ritz B. (2005). Clinical characteristics in early Parkinson’s disease in a central california population-based study. *Mov. Disord.* 20 1133–1142. 10.1002/mds.20513 15954133 PMC3643967

[B31] Kelly V. E. Eusterbrock A. J. Shumway-Cook A. (2012). A review of dual-task walking deficits in people with parkinson’s disease: motor and cognitive contributions, mechanisms, and clinical implications. *Parkinsons Dis.* 2012 918719. 10.1155/2012/918719 22135764 PMC3205740

[B32] Lord S. Rochester L. Hetherington V. Allcock L. M. Burn D. (2010). Executive dysfunction and attention contribute to gait interference in “off” state Parkinson’s Disease. *Gait Posture* 31 169–174. 10.1016/j.gaitpost.2009.09.019 19896382

[B33] Luo L. Andrews H. Alcalay R. N. Poyraz F. C. Boehme A. K. Goldman J. G. (2019). Motor phenotype classification in moderate to advanced PD in BioFIND study. *Parkinson. Relat. Disord.* 65 178–183. 10.1016/j.parkreldis.2019.06.017 31255537 PMC6774826

[B34] Markello R. D. Shafiei G. Tremblay C. Postuma R. B. Dagher A. Misic B. (2021). Multimodal phenotypic axes of Parkinson’s disease. *NPJ Parkinsons Dis.* 7 6. 10.1038/s41531-020-00144-9 33402689 PMC7785730

[B35] Marras C. Chaudhuri K. R. (2016). Nonmotor features of Parkinson’s disease subtypes. *Mov. Disord.* 31 1095–1102. 10.1002/mds.26510 26861861

[B36] Mehrholz J. Kugler J. Storch A. Pohl M. Hirsch K. Elsner B. (2016). Treadmill training for patients with Parkinson disease. An abridged version of a Cochrane Review. *Eur. J. Phys. Rehabil. Med.* 52 704–713. 26940123

[B37] Meireles J. Massano J. (2012). Cognitive impairment and dementia in Parkinson’s disease: clinical features, diagnosis, and management. *Front. Neurol.* 3:88. 10.3389/fneur.2012.00088 22654785 PMC3360424

[B38] Miyai I. Fujimoto Y. Ueda Y. Yamamoto H. Nozaki S. Saito T. (2000). Treadmill training with body weight support: Its effect on Parkinson’s disease. *Arch. Phys. Med. Rehabil.* 81 849–852. 10.1053/apmr.2000.4439 10895994

[B39] Morris M. E. Iansek R. Matyas T. A. Summers J. J. (1994). The pathogenesis of gait hypokinesia in Parkinson’s disease. *Brain* 117 1169–1181. 10.1093/brain/117.5.1169 7953597

[B40] Moseley A. M. Stark A. Cameron I. D. Pollock A. (2005). Treadmill training and body weight support for walking after stroke. *Cochrane Database Syst. Rev.* 4 CD002840. 10.1002/14651858.CD002840 16235304

[B41] Mukherjee M. Siu K.-C. Katsavelis D. Fayad P. Stergiou N. (2011). The influence of visual perception of self-motion on locomotor adaptation to unilateral limb loading. *J. Motor Behav.* 43 101–111. 10.1080/00222895.2010.548420 21347952

[B42] Muslimovic D. Post B. Speelman J. D. Schmand B. de Haan R. J. (2008). Determinants of disability and quality of life in mild to moderate Parkinson disease. *Neurology* 70 2241–2247. 10.1212/01.wnl.0000313835.33830.80 18519873

[B43] Nakamura T. Meguro K. Sasaki H. (1996). Relationship between falls and stride length variability in senile dementia of the Alzheimer Type. *Gerontology* 42 108–113. 10.1159/000213780 9138973

[B44] Nanhoe-Mahabier W. Snijders A. H. Delval A. Weerdesteyn V. Duysens J. Overeem S. (2013). Split-belt locomotion in Parkinson’s disease with and without freezing of gait. *Neuroscience* 236 110–116. 10.1016/j.neuroscience.2013.01.038 23370318

[B45] Nasreddine Z. S. Phillips N. A. Bédirian V. Charbonneau S. Whitehead V. Collin I. (2005). The Montreal Cognitive Assessment, MoCA: a brief screening tool for mild cognitive impairment. *J Am Geriatr Soc.* 53 695–699. 10.1111/j.1532-5415.2005.53221.x 15817019

[B46] Nieuwhof F. Bloem B. R. Reelick M. F. Aarts E. Maidan I. Mirelman A. (2017). Impaired dual tasking in Parkinson’s disease is associated with reduced focusing of cortico-striatal activity. *Brain* 140 1384–1398. 10.1093/brain/awx042 28335024

[B47] Nutt J. G. (2016). Motor subtype in Parkinson’s disease: different disorders or different stages of disease? *Mov. Disord.* 31 957–961. 10.1002/mds.26657 27226220

[B48] Orgeta V. McDonald K. R. Poliakoff E. Hindle J. V. Clare L. Leroi I. (2020). Cognitive training interventions for dementia and mild cognitive impairment in Parkinson’s disease. *Cochrane Database Syst. Rev.* 2 CD011961. 10.1002/14651858.CD011961 PMC7043362 32101639

[B49] O’Shea S. Morris M. E. Iansek R. (2002). Dual task interference during gait in people with Parkinson disease: effects of motor versus cognitive secondary tasks. *Phys. Ther.* 82 888–897. 10.1093/ptj/82.9.888 12201803

[B50] Pagonabarraga J. Kulisevsky J. (2012). Cognitive impairment and dementia in Parkinson’s disease. *Neurobiol. Dis.* 46 590–596. 10.1016/j.nbd.2012.03.029 22484304

[B51] Patla A. E. Niechwiej E. Davies T. C. (2004). Obstacle avoidance during locomotion using haptic information in normally sighted humans. *Exp. Brain Res.* 155 173–185. 10.1007/s00221-003-1714-z 14770274

[B52] Peters S. Handy T. C. Lakhani B. Boyd L. A. Garland S. J. (2015). Motor and visuospatial attention and motor planning after stroke: considerations for the rehabilitation of standing balance and gait. *Phys. Ther.* 95 1423–1432. 10.2522/ptj.20140492 25929533 PMC4595814

[B53] Petzinger G. M. Fisher B. E. McEwen S. Beeler J. A. Walsh J. P. Jakowec M. W. (2013). Exercise-enhanced neuroplasticity targeting motor and cognitive circuitry in Parkinson’s disease. *Lancet Neurol.* 12 716–726. 10.1016/S1474-4422(13)70123-6 23769598 PMC3690528

[B54] Pickering R. M. Grimbergen Y. A. M. Rigney U. Ashburn A. Mazibrada G. Wood B. (2007). A meta-analysis of six prospective studies of falling in Parkinson’s disease. *Mov. Disord.* 22 1892–1900. 10.1002/mds.21598 17588236

[B55] Plotnick M. Giladi N. Dagan Y. Hausdorff J. M. (2011). Postural instability and fall risk in Parkinson’s disease: impaired dual tasking, pacing, and bilateral coordination of gait during the “ON” medication state. *Exp. Brain Res.* 210 529–538. 10.1007/s00221-011-2551-0 21279632

[B56] Podsiadlo D. Richardson S. (1991). The timed “Up & Go”: a test of basic functional mobility for frail elderly persons. *JAmGeriatrSoc.* 39 142–148. 10.1111/j.1532-5415.1991.tb01616.x 1991946

[B57] Pohl M. Rockstroh G. Rückriem S. Mrass G. Mehrholz J. (2003). Immediate effects of speed-dependent treadmill training on gait parameters in early Parkinson’s disease. *Arch. Phys. Med. Rehabil.* 84 1760–1766. 10.1016/s0003-9993(03)00433-7 14669180

[B58] Pont-Sunyer C. Hotter A. Gaig C. Seppi K. Compta Y. Katzenschlager R. (2015). The onset of nonmotor symptoms in Parkinson’s disease (the ONSET PD study). *Mov. Disord.* 30 229-–37. 10.1002/mds.26077 25449044

[B59] Rahman S. Griffin H. J. Quinn N. P. Jahanshahi M. (2008). Quality of life in Parkinson’s disease: the relative importance of the symptoms. *Mov. Disord.* 23 1428–1434. 10.1002/mds.21667 18543333

[B60] Rochester L. Baker K. Hetherington V. Jones D. Willems A.-M. Kwakkel G. (2010). Evidence for motor learning in Parkinson’s disease: Acquisition, automaticity and retention of cued gait performance after training with external rhythmical cues. *Brain Res.* 1319 103–111. 10.1016/j.brainres.2010.01.001 20064492

[B61] Rovini E. Maremmani C. Cavallo F. (2017). How wearable sensors can support Parkinson’s disease diagnosis and treatment: a systematic review. *Front. Neurosci.* 11:555. 10.3389/fnins.2017.00555 29056899 PMC5635326

[B62] Schaafsma J. D. Giladi N. Balash Y. Bartels A. L. Gurevich T. Hausdorff J. M. (2003). Gait dynamics in Parkinson’s disease: relationship to Parkinsonian features, falls and response to levodopa. *J. Neurol. Sci.* 212 47–53. 10.1016/s0022-510x(03)00104-7 12809998

[B63] Seuthe J. D’Cruz N. Ginis P. Weisser B. Berg D. Deuschl G. (2019). Split-belt treadmill walking in patients with Parkinson’s disease: A systematic review. *Gait Posture* 69 187–194. 10.1016/j.gaitpost.2019.01.032 30771729

[B64] Schumway-Cook A. Brauer S. Woollacott M. (2000). Predicting the probability for falls in community-dwelling older adults using the timed up & go test. *Phys. Ther.* 80 896–903. 10.1093/ptj/80.9.896 10960937

[B65] Smith A. (1982). *Symbol Digit Modalities Test (SDMT). Manual (Revised).* Los Angeles, CA: Western Psychological Services.

[B66] Sofuwa O. Nieuwboer A. Desloovere K. Willems A.-M. Chavret F. Jonkers I. (2005). Quantitative gait analysis in Parkinson’s disease: comparison with a healthy control group. *Arch. Phys. Med. Rehabil.* 86 1007–1013. 15895349 10.1016/j.apmr.2004.08.012

[B67] van Rooden S. M. Colas F. Martínez-Martín P. Visser M. Verbaan D. Marinus J. (2011). Clinical subtypes of Parkinson’s disease. *Mov. Disord.* 26 51–58. 10.1002/mds.23346 21322019

[B68] Vitório R. Lirani-Silva E. Barbieri F. A. Raile V. Stella F. Gobbi L. T. B. (2013). Influence of visual feedback sampling on obstacle crossing behavior in people with Parkinson’s disease. *Gait Posture* 38 330–334. 10.1016/j.gaitpost.2012.12.019 23347768

[B69] Wang X. Chen L. Zhou H. Xu Y. Zhang H. Yang W. (2021). Enriched rehabilitation improves gait disorder and cognitive function in Parkinson’s disease: a randomized clinical trial. *Front. Neurosci.* 15:733311. 10.3389/fnins.2021.733311 34924926 PMC8674725

[B70] Wojtala J. Heber I. A. Neuser P. Heller J. Kalbe E. Rehberg S. P. (2019). Cognitive decline in Parkinson’s disease: the impact of the motor phenotype on cognition. *J. Neurol. Neurosurg. Psychiatry* 90 171–179. 10.1136/jnnp-2018-319008 30297519

[B71] Yang Y.-R. Cheng S.-J. Lee Y.-J. Liu Y.-C. Wang R.-Y. (2019). Cognitive and motor dual task gait training exerted specific training effects on dual task gait performance in individuals with Parkinson’s disease: a randomized controlled pilot study. *PLoS One* 14:e0218180. 10.1371/journal.pone.0218180 31220121 PMC6586283

[B72] Yogev G. Giladi N. Peretz C. Springer S. Simon E. S. Hausdorff J. M. (2005). Dual tasking, gait rhythmicity, and Parkinson’s disease: which aspects of gait are attention demanding? *Eur. J. Neurosci.* 22 1248–1256. 10.1111/j.1460-9568.2005.04298.x 16176368

